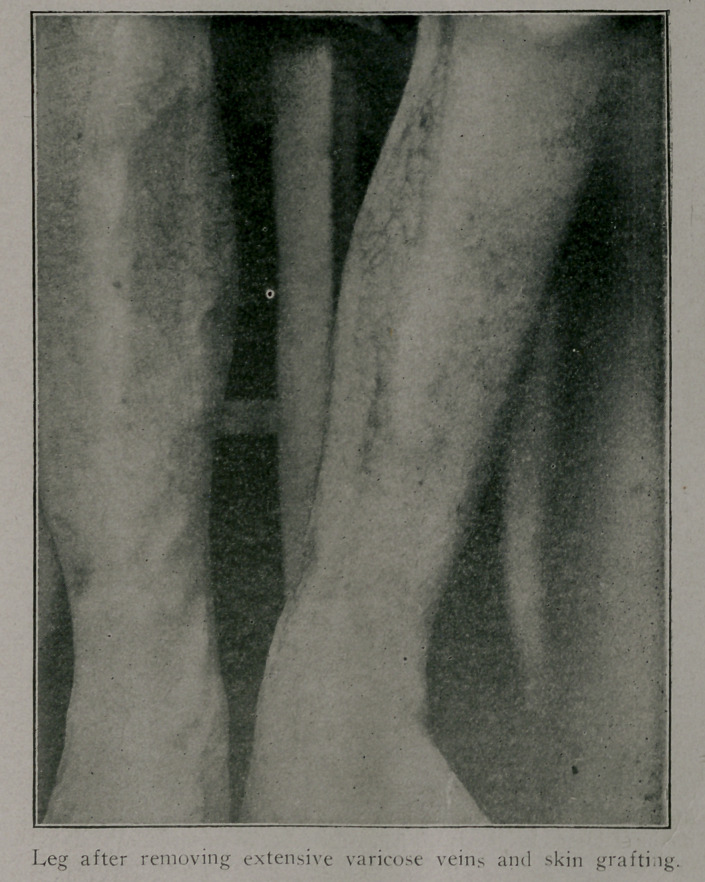# A Report of Wednesday’s Surgical Clinic in the Atlanta School of Medicine**The operations included in this report occurred during the sessions of* 1910-’11 *and* 1911-’12. *No private patients are included*.

**Published:** 1912-10

**Authors:** Edward G. Jones, Dean F. Winn

**Affiliations:** Atlanta, Ga.; Atlanta, Ga.


### Journal-Record of Medicine

Successor to Atlanta Medical and Surgical Journal, Established 1855
and Southern Medical Record, Established 1870.

OWNED BY THE ATLANTA MEDICAL JOURNAL CO.

**Published Monthly**

*Official Organ Fulton County Medical Society, State Examining Board, Presbyterian Hospital, Atlanta, Birmingham and Atlantic Railroad Surgeons' Association, Chattahoochee Valley Medical and Surgical Association, Etc.*

EDGAR G. BALLENGER, M. D., Editor.
BERNARD WOLFF, M. D., Supervising Editor.
A. W. STIRLING, M. D., C. M., D. P. H., J. S. HURT, B. Ph., M. D.
GEO. M. NILES, M. D., W. J. LOVE, M. D., (Ala.); Associate Editors.
E. W. ALLEN, Business Manager.

COLLABORATORS

Dr. W. F. WESTMORLAND, General Surgery.
F. W. McRAE, M. D., Abdominal Surgery.
H. F. HARRIS, M. D., Pathology and Bacteriology.
E. B. BLOCK, M. D., Diseases of the Nervous System.
MICHAEL HOKE, M. D., Orthopedic Surgery.
CYRUS W. STRICKLER, M. D., Legal Medicine and Medical Legislation.
E. C. DAVIS, A. B., M. D., Obstetrics.
E. G. JONES, A. B., M. D., Gynecology.
R. T. DORSEY, Jr., B. S. M. D., Medicine.
L. M. GAINES, A. B., M. D., Internal Medicine.
GEO. C. MIZELL, M. D., Diseases of the Stomach and Intestines.
L. B. CLARKE, M. D., Pediatrics.
EDGAR PAULIN, M. D., Opsonic Medicine.
THEODORE TOEPEL, M. D., Mechano Therapy.
R. R. DALY, M. D., Medical Society.
A. W. STIRLING, M. D., etc., Diseases of the Eye, Ear, Nose and Throat.
BERNARD WOLFF, M. D., Diseases of the Skin.
E. G. BALLENGER, M. D., Diseases of the Genito-Urinary Organs.

VOL. LIX. OCTOBER 1912 NO. 7

### A REPORT OF WEDNESDAY'S SURGICAL CLINIC IN THE ATLANTA SCHOOL OF MEDICINE*

BY EDWARD G. JONES, A.B., M.D., AND DEAN F. WINN, M.D.,

ATLANTA, GA.

We have grouped the cases with reference to the various regions of the body. In some instances we have given the operation

---

**The operations included in this report occurred during the sessions of 1910-'11 and 1911-'12. No private patients are included.*

in detail. One's inability to keep track of clinic patients, of course, discounts the value of statements about ultimate results.

### HEAD AND NECK.

*Cyst of Parotid.* There was a small cyst in the paroti.l gland containing about a drachm of mucoid substance. A probe was passed into Steno's duct disproving the possibility of a stricture as a cause of the collection. The cavity was opened in a direction parallel to the facial nerve fibers, swabbed out with phenol and alcohol and drained. The sac was not dissected out because it was evident that it could be destroyed without incurring risk to the facial nerve.

*Osteoma Inferior Maxilla.* This woman had a benign bony tumor which occupied, as can be seen by the accompanying cut, all of the lower jaw except the ascending rami. X-ray supported the diagnosis as to the nature of the growth. The tumor had been growing for ten years and extended some two inches backward below the floor of her mouth. The patient could, with difficulty, close her mouth by rolling her lower lip over her teeth which extended two inches beyond the normal line. Subperiosteal resection was performed, the periosteum being left with the idea of providing opportunity for new bone formation. Her jaw was practically useless and a great inconvenience to her before the tumor was removed.

A word of caution should be coupled with the statement that this patient has had no recurrence. *A very large percentage of tumors of both maxillae are malignant at any age, and their early removal constitutes the patient's only hope.*

*Harelip.* There was one case of harelip. All the tension on the lip was relieved by extensively separating it from the bony structures. The sides of the defect were sutured together after paring off, everting and making redundant the vermilion border. We are accustomed to insert all sutured subcutaneously and to tie the stay sutures on the mucous (inner) surface to avoid stitch hole scars on the skin surface. The flare of the nostril was corrected by shotted silver wire passed underneath its floor.

*Tracheotomy.* This patient gave a history of having aspirated a piece of straw and had great difficulty in breathing. Competent larynologists could find no foreign body in the upper air passages. Tracheotomy was performed and a tube inserted, no

Photograph of patient before removing large osteoma of the lower jaw.

foreign body being found. The tube relieved the dyspnoea, however, this fact indicating that the obstruction was above the tracheotomy opening. Further laryngoscopic examination both through the artificial opening and in the usual way failed to reveal anything except a moderate edema. The boy was allowed to go home with the tube in place; we have heard that he died two or three months subsequently—under circumstances about which we do not know.

A foreign body in the air passages presents a most serious situation. This is especially so if it will not cast an X-ray shadow and is of such a nature as not to be recognized when touched with an instrument. Bronchoscopy through a tracheal opening is a valuable advance, though its successful utilization is beset with many restrictions.

Nor does a radiograph of itself, when revealing the body, make its removal an easy task.

Two years ago (Surgery, Gynecology and Obstetrics, May 1910) Francis Huber and Robert T. Morris respectively, reported the removal of a tack and pin from the bronchi by guiding the forceps through a fluoroscopic screen.

*Cervical Adenitis.* There were two cases of simple pyogenic infection of the glands in the neck, submaxillary and suboccipital. Both were drained under cocaine anesthesia.

One patient had tuberculous glands on both sides of the neck. The submaxillary and submental glands were the only ones affected. They were exposed through a transverse incison in a normal crease of the skin, the incision extending between points a short distance in front of the angles of the jaw. The glands and the gland-bearing fascia were dissected out. It is well to preserve the attachment of the glands to the fascia, as this facilitates their removal, and to begin the dissection in the middle line, removing the glands and fascia on either side separately. The hypoglossal nerves are the only important structures to avoid, and one of these may be injured without doing great harm. Little inconvenience follows the removal of the submaxillary salivary glands. Usually their removal is necessary on account of their close connection with the infected lymph nodes. In this case caseous material escaped from one of the glands and the area was swabbed with tincture iodine. The cut edges of the platysma, which had been reflected with the skin, were carefully sutured in order to prevent separation in the scar.

*Epithelioma of Forehead.* The growth was excised and the diagnosis of malignancy confirmed by microscopical section. The patient returned home before the wound healed and reports that there is still some raw surface.

Osteoma inferior maxilla. (upper aspect).

*Cancer of Lip.* This patient exhibited a growth on the lower lip and involvement of the submental and submaxillary glands. The growth was excised by a V shaped incision. As represented in the accompanying cut, the mucous membrane of the cheek was swung in to form the new lip. The submaxillary and submental glands were removed through a traverse incision in the neck Stab wound was made for drainage below the neck incision.

Removal of the lesion on the lip by paste, caustics or by simply snipping out the growth will not cure the patient.

Extension by the lymphatics occurs slowly and by only one route:- the submental and submaxillary glands. These glands are completely accessible. Their removal should receive the same care as is exercised in dealing with the axillary glands in cancer of the breast.

Swellings in this region of the neck should call for questions regarding a past history of ulcer of the lip.

### CHEST.

*Chronic Empyema.* The thorax had been drained sometime previously and a sinus persisted. One rib was resected and the sinus excised. The patient made an ideal recovery.

*Lipoma.* This was a large tumor in the scapular region and was excised under cocaine.

*Tumors of the Breast.* There were three patients with tumors of the breast. One of these was apparently innocent, but inasmuch as all female breast tumors are presumably malignant, the tumor was excised and sent for diagnosis to a pathologist who verified the opinion that it was a benign growth.

The other two cases were carcinomata. Radical amputation was performed. Closure was effected by the Jackson method in one case, and one case required subsequent skin grafting. There is no information at hand as to recurrence or death.

Respecting tumors of the female breast we are governed by the following facts:—

1. *Eighty percent* are malignant when they appear: an additional *ten percent* become malignant if let alone.

2. All are to be considered malignant until the contrary is definitely proved.

3. After 35 more women die of cancer of all organs than of tuberculosis.

4. After the menopause the reasonably possible tumors are *cancer, sarcoma, and cysts,* with the probability greatly in favor of cancer.

5. Retraction of the nipple is pathognomonic if present (excluding previous mastitis) *but may not be present in cancer.*

6. Under no circumstances should a diagnosis wait on the appearance of any symptom whatever; the presence of a "lump" shouts aloud for immediate action.

Cancer, nowhere, is announced by *pain;* it is a great pity it is not ushered in like toothache.

Palpable axillary glands should *on no account* be waited for; when they can be felt it is usually too late to save the patient.

Osteoma lower jar (Anterior view).

Nor is the general health any aid to diagnosis.

7. The prognosis depends (in order) upon:-

(a). Duration of tumor.

(b). Age of patient (older patients most hopeful).

(c). Kind of cancer (adeno-carcinoma probably least malignant).

8. There is no treatment except radical removal.

9. Even bad surgery done early is better than good surgery done late.

### ABDOMEN.

*Tuberculous Peritonitis.* Both of these patients were operated on under local anesthetic. Both had the ascitic type of the disease. The fluid was evacuated through a median incision and a split tube drain inserted. Our experience with the cure of this disease by simple celiotomy agrees with that of other operators, though we have seen no adequate reason for the success attending this method of treatment. The patients were subsequently placed upon the usual dietetic and hygienic treatment.

*Fecal Fistula.* Ordinarily such fistulae will heal spontaneously if given time, but occasionally they will not. One of these cases was of comparatively short duration following an incision for an appendix abscess. After sealing the external opening of the fistula the tract was isolated through a nearby incision and removed. Manipulation of the fistulous tract and closure of the hole in the cecum were accomplished without contamination of the surrounding viscera by the careful use of gauze pads.

The second patient presented a discharging sinus at the crest of the ilium through Petit's triangle. The patient had been previously operated on, and a piece of bone was said to have been removed. Exploration revealed the fact that the tract connected with the posterior wall of the cecum behind the peritoneum, having evidenly been caused by a ruptured postcecal abscess. There was no bony involvement.

*Hernia.* There were two right inguinal and two double inguinal herniae. One of the right side cases also had hydrocele on the same side. In one case of double hernia the bladder formed

Mehod of removing submaxillary and submental glands and fascia in cancer of lip and tuberculous adenitis.

a part of the contents of the sac. This case had been previously operated on and had probably had infection, for considerable destruction of tissue had occurred. It was necessary to utilize the fascia lata and the sheath of the rectus to repair the canal. On all of the patients the general principles of Bassini's (Marcy's) operation were followed with such modifications as the individual cases required.

We are impressed that in the average oblique inguinal hernia the *characteristic and material defect* is too scant attachment of the internal oblique and transversalis inward along Poupart's ligament (Ferguson). While the usual additional measures have been made use of, this defect has in all operations received prime consideration in the repair; and in a series of operations of some extent there has been no known recurrence.

Success or failure will commonly be determined by the thoroughness with which all unnecessary tissue is cleared from the canal and an anatomical defect existing in a large percentage of individuals is subsequently corrected.

Attempts to cure at or near the external ring will be futile.

*Appendicitis.* Twenty patients were operated on for appendicitis. One patient had a large well walled-off abscess, with the appendix lying in such a position as to make its removal justifiable. Had there been any doubt about the wisdon of removing the appendix it would have been left for future developments. We believe that an operator rarely makes the mistake of being too conservative in this respect. This patient died apparently of embolism and was the only death occurring in this series.

One patient had a gangrenous perforated appendix. The clinical picture in this case was quite deceptive in that the attack appeared to be subsiding and the patient to be getting well. Two appendices contained pus and fecal concretions but were not ruptured. Two cases were of the simple catarrhal type; two were in the subsiding stage; and four were examples of chronic appendicitis. In all of these last, indigestion was the most prominent symptom. Lane's kink was noted in one instance. The lumen was obliterated in one case and the appendix buried under old adhesions. Eight appendices were removed in conjunction with other operations. These cases all showed some definite pathology; we did not make it a practice to remove the appendix simply because the abdomen was opened, though we really doubt the wisdom of this plan in persons in the appendicitis age.

It is to be regretted that the following facts cannot be said to have been generally accepted as true by the profession :-

1. Nearly all attacks called "acute indigestion" in persons under forty are really attacks of appendicitis.

2. Very many persons suffering from "chronic indigestion" will be cured by appendix operations.

Incision for cancer of the lip. (Esmarch and Kowalzig).

3. Nearly every person with appendicitis operated on early by a competent surgeon will recover.

4. The majority of persons will survive an attack of appendicitis without operation; but one cannot possibly tell in advance which patient *will not thus survive.*

*Cholelithiasis.* This patient had a typical history of gall stones. A single stone was removed from the cystic duct and the gall bladder drained.

Attacks of "acute indigestion" in women past 40, especially if they have borne children, are usually gall stone attacks.

*Uterine Fibroids.* Twenty patients had fibroid tumors of the uterus and hysterectomy was performed upon all of them. Both ovaries were removed in ten cases; only one ovary in six cases; both ovaries were left in two cases; and there is no record regarding two cases. The appendix was removed in eight cases. Case No. 12 had a large intraligamentous tumor on the left side which had pushed the ureter in front of it. Case No. 14 had enormous fibroids surrounded by dense adhesions. Immense vessels passed from the omentum to the tumors. In case No. 18 the tumor was bisected on account of the difficulty in raising it. Two tumors enroached on the bladder. Case No. 20 had a pelvic abscess in addition to fibroids. The abscess was drained through the vagina and hysterectomy performed three weeks later.

Supravaginal hysterectomy was the usual procedure. The cervix was supported by suturing the round ligaments to it. Pathologic ovaries are commonly found with uterine fibroids, especially in clinic patients who have usually had diseased appendages and who have frequently permitted the tumor to assume large proportions before submitting to surgical intervention; but whenever it appeared wise at least one or a part of one ovary was left, especially if the patient had not passed the menopause However, we endeavored not to allow our judgment with reference to diseased ovaries to become obscured in the effort to avoid the production of an artificial menopause.

*Ovarian Cysts.* Four patients were operated on. One had a cyst weighing about 75 pounds which was removed together with the uterus. Another had a large cyst which had been aspirated several times, adhesions resulting which greatly increased the difficulty of the operation. A simple cyst, which had practically destroyed the ovary, was discovered during an operation for appendicitis and was removed. One patient, on whom exploratory laparotomy was done, had a malignant degenerating papillomatous cyst which had progressed too far to warrant hope of a cure by operation. One patient required resection of one ovary for hematoma.

*Abscess of the Liver.* The patient was a man, aged 50. He gave a history of chronic dysentery but had not suffered from it for the past ten years. The liver extended two inches below the costal margin. A fluctuating tumor presented in the region of the gall bladder. The abdomen was opened over the tumor and an abscess of the liver exposed. Attempt was made to suture the liver to the abdominal incision before opening the abscess, but was unsuccessful owing to the thinness and the friability of the abscess wall. Soiling of the abdomen was prevented by packing gauze pads around the incision. The abscess cavity was drained after evacuating about a pint of thick pus. The patient made an uneventful recovery.

### Vagina and Rectum.

*Pelvic abscess.* This patient had a fibroid tumor in addition to a pelvic abscess. Her vitality had been extremely lowered by hemorrhage and septic absorption. Colpotomy was performed and later supravaginal hysterectomy.

*Hemorrhoids.* One patient was operated on. Hemorrhoids were removed with clamp and cautery. We prefer this method to that of suturing because it is rarely followed by post-operative pain. Ordinary plumber's soldering irons are entirely satisfactory and have the advantage of always being in working order.

We cannot agree with the somewhat prevalent idea that an operation for hemorrhoids is a more or less trivial matter and "can be done by anybody." We must confess that the getting of a perfectly satisfactory result gives us more advance concern than in case of many more formidable operative undertakings. Yet a careful study of the local condition and an intelligently conducted operation will almost always result ideally and provoke high praise from grateful patients.

*Stricture of the Rectum.* This was an operation of necessity and was undertaken with the knowledge that the patient was a poor risk on account of an aortic regurgitation. Unusual precautions were taken about the anesthesia, but the patient died suddenly before the operation was begun.

### GENITO-URINARY.

*Lymphangioma of Scrotum.* The patient was above fifty years of age. He had an oval mass about three inches in diameter in the lower end of his scrotum. The scrotum was greatly elongated, the tumor swinging between the patients knees. The condition had been present some ten years. The skin on the inner side of one knee and on both sides of the adjacent parts of the scrotum had been barked off by the constant rubbing of the parts. The patient was a farmer and had continued to plough and attend to his other duties for many years after the growth had reached its present development. Another mass occupied the region of the perineum, extending into both ischio-rectal fossae and involving the overlying skin. The two masses seemed to have been separated by the weight of the lower portion. He had no complaint other than the inconvenience caused by the tumor hanging between his knees. The entire growth, together with the scrotum and testicles, was removed. Complete closure of the incision on the buttocks could not be affected and subsequent skin grafting was necessary. Microscopical examination showed the growth to be a lymphangioma.

*Hydrocele.* There were four cases of hydrocele occurring on one side only and one case of double hydrocele. Eversion of the sac was the operation used in all the cases.

*Varicocele.* Two cases of varicocele, left side, were operated on, the veins being resected through a scrotal incision in both instances. One of these patients also required amputation of the scrotum on account of elongation.

*Stricture of Urethra.* This case was a man 60 years old who had had for five years an almost impassable stricture of the deep urethra and two urinary fistulae opening on the perineum. With prolonged effort to filiform sound was passed. External urethrotomy was done and catheter drainage instituted through the urethra. The fistulae were closed to the median line and were incised, curetted and packed with iodoform gauze. Six months after the patient went home he writes that he is entirely well and "keeps a row ahead of a string of niggers choppin' cotton."

*Vesical Calculus.* Suprapubic cystotomy was performed one time, a large stone, about the size of a hen egg, being removed.

*Epididymitis.* (Drs. Winn and Wagnon). This case is of interest because operative measures were followed by such happy results after the ordinary line of treatment had failed. The condition was of gonorrhoeal origin. Further experience with the operative treatment of epididymitis leads us to believe that were more cases so treated convalesence would be greatly shortened in the majority of them. The operation done was after the method of Hagner and has been endorsed by the leading urologists.

Deliver the testicle and epididymis through an incision in the scrotum. Open the tunica vaginalis and evacuate the fluid. (There is nearly always acute hydrocele.) The fluid is sometimes tinged with blood, though commonly it is straw-colored and clear. The epididymis is hard, indurated and swollen. Usually the globus minor is the part most intensely inflammed. Incise the tough, fibrous capsule covering the globus minor; if pus does not escape, explore gently with a probe and compress the parts. Usually from a few drops to a drachm of pus will escape. Explore likewise the remainder of the epididymis. The testicle is rarely ever involved. The tunica may be closed with catgut or trimmed off and everted. Wash the parts with bichloride of mercury 1-1000 and salt solution and replace them within the scrotum. Close the skin after instituting drainage through a stab wound in the lower part of the scrotum; this allows the incision to close without becoming infected. Remove the drain at the end of 48 hours.

The patient is absolutely free from pain when he recovers from the anesthetic.

The systemic symptoms are promptly relieved.

The danger to the patient is slight if proper care is exercised.

The infiltration disappears rapidly. The epididymis resumes its normal size and consistency.

The patient can leave the hospital in three or four days after operation and resume his work within a week.

The danger of permanent injury to the testicle and epididymis is lessened. We believe that there is not so much damage done to the tubules by exploration as is done by other methods of treatment where the infiltration never entirely absorbs but remains as a fibrous nodule with a tendency to occlude the tubules and annoy a patient who is inclined to be neurasthenic.

We have had no recurrences.

We have not failed to find pus in a single instance, though only about 80% are said to have pus. If there was pus confined in any other locality no one would question the wisdom of securing ample drainage.

*Hypospadias.* This case was of the balanitic type and was repaired after Beck's method.

### Lower Extremity.

*Varicose Veins.* The principle of the surgical treatment of varicose veins of the leg is the transference of the venous circulation from the superficial to the deep veins. One must remember that varicosed superficial veins may be an effort of nature to counteract the effect of some obstruction in the deep veins. In doubtful cases it is well to keep an elastic bandage on the limb for about a week; if this gives comfort one may be fairly certain that the deep veins are capable of functionating properly.

Two cases of varicose veins occurred in this series. One is interesting because of its rarity. The patient appeared for treatment with the statement that he had a few minutes before begun to bleed from his right leg, his attention having been attracted by the dampness of his sock. He complained of no pain but was nauseated and weak. He denied any history of trauma. Examination revealed an opening in the skin, about the size of an ordinary match head, over the crest of the tibia at the junction of the middle and lower thirds of the leg, from which blood was welling up profusely. The leg was not bruised and there was no inflammation about the wound. There was slight swelling of the

Leg after removing extensive varicose veins and skin grafting.

ankle and a few varicose veins were visible. Hemorrhage was stopped promptly by pressure over the wound and bandaging. This man claimed to have been unaware of the fact that he had varicose veins until this spontaneous rupture occurred.

The accompanying cut shows the second case. The superfical veins on the anterior and inner surface of the leg were dissected out, the incision beginning just below the knee and extending to the foot of the toes. The wound unfortunately became infected and it was necessary to remove all the stitches. After the wound had become covered with clean granulations, skin was grafted under cocaine from the outer side of the thigh by the Reverdin method. Practically all the grafts lived. None of the grated skin has since broken down. There have been no more ulcers formed on the leg since the operation, six months ago. An ulcer, which existed at the time of operation and which was curetted and grafted, remains well. Normal sensation is gradually appearing in the grafted area, patient having complained of numbness for about two months after operation. The leg does not swell. Before excising the veins it was demonstrated that the Trendelenburg operation (ligation of the external saphenous vein just below the saphenous opening) would not suffice. This test is made by elevating the leg and allowing the veins to empty themselves. When this is accomplished make pressure over the saphenous opening and lower the leg. If the veins become filled while this pressure is being made, ligation of the external saphenous vein is not the best procedure.

*Lipoma.* One patient had a lipoma of the thigh which was excised.

*Ulcer Gluteal Region.* This case had a scar, caused by an old burn, extending from the left lumbar region to the lower half of the thigh. At a point just posterior to the grafted trochanter, an ulcer, 1-4 inch deep and as large as a silver dollar, had existed for five years. The ulcer was acutely sensitive. It was curetted and the margins cut away. At a later date, when granulations had formed, the area was skin grafted (Reverdin method, local anesthesia). The ulcer healed promptly.

*Sarcoma Femur.* This patient, a young man, had a rapidly growing tumor of the femur which was evidently sarcomatous. Spontaneous fracture had occurred. Hip-joint amputation was performed with practically no loss of blood and without any great degree of shock. Six months later there is no sign of a recurrence and the patient weighs twenty pounds more than he did prior to removal of the leg.

*Osteo-myelitis femur.* The middle two fourths of the femur was involved, with two sinuses opening on the inner and anterior aspect of the thigh. The diseased bone was removed and the wound allowed to granulate. The sinuses were curetted and drained.

MISCELLANEOUS.

*Salvarsan.* (Drs. Winn and Wagnon). This is one of a series of a large number of doses of 606 given during the past year. A review of the patients, most of whom we have been able to keep in touch with, leads us to endorse it enthusiastically as an important adjunct to the treatment of syphilis. In not a single case did it fail to give at least temporary improvement in the symptoms, many of the cases bearing out the marvelous results reported by others. A large percentage required a second dose on account of recurrence of symptoms, and a few a third dose, but we have made it a practice to always repeat salvarsan whenever the patients would permit it even though they presented no signs of a return of symptoms. With the exception of one case these patients have experienced no discomfort following the administration, other than the usual diarrhoea and nausea, and a great many cases reported no unpleasant symptoms at all.

The one exception was an Italian, aged 38, stonemason, who had been treated in Italy by hypodermic injection of mercury and iodide of potash by mouth. He still complained of persistent headache and had enlarged inguinal glands. 606 was administered intravenously and the patient told to go home immediately. Instead, he took a long car ride and developed diarrhoea, nausea and abdominal cramps before reaching home. He was seen five hours after taking the dose and presented a typical picture of acute arsenic poisoning. During the next twenty-four hours morphine was required to relieve his intense abdominal pain, and and although he drank a copious amount of lithia water and was given spartein and acetate of potash, he excreted only three drachms of urine during this time. We do not believe he had enough morphine to depress his kidney action to such an extent. He was able to return to work in ten days, though a severe herpes on his face and lips continued for about a week longer. Second dose was refused. The glands have disappeared and he is without symptoms. He has a negative Wassermann. His wife has since given birth to a normal baby.

In all cases we have followed the administration of 606 with the ordinary treatment of mercury and potassium iodide.

Intravenous injection has been the only method employed. We have never, save in two instances, resorted to the uncertain procedure of inserting a sharp needle into the vein through the skin, but have preferred the more surgical method of exposing the vein and inserting a blunt canula. We have never had a patient to object to the scar. In not a single instance have we had swelling, infection, sloughing, etc., at the site of injection.

*Direct Transfusion of Blood.* Feeling that experience upon lower animals is essential to enable one to properly carry out this delicate technique, we presented to the class some of the various methods then in use, using dogs for the work. Vein to vein, and vein to artery anastomosis, the use of Crile's canulae and petrolatum-coated glass canulae, and vein transplantation were demonstrated.

*Skin Grafting.* There were five cases of skin grafting; two ulcers of the buttock, one wound on dorsum of foot caused by snake bite, one infected wound of the leg and one area on the breast following amputation. In all these cases, cocaine anesthesia was used. The method employed was that of Reverdin. None of the cases lost enough of the grafts as to require a second operation, and in most of them about 75% of the grafts lived. Autogenous grafts were used in all cases because they are much more apt to live than is skin taken from another individual.

Iodine sterilization of the skin seemed not to affect the vitality of the grafts.

Healing was greatly promoted by the use of an 8% ointment of Scarlet Red which was applied as soon as it became evident that the grafts would live. Scarlet Red undoubtedly has a strong stimulating effect upon epitheliation. Our experience with its use in this and other series of cases has shown that, when properly used, it caused an average growth of epithelium of from 3 to 4 mm. in 48 hours, but that this rate of stimulation decreases after repeated applications. The skin thus formed is thick, unlikely to break down and freely movable over the underlying tissues, and very little contraction takes place in the wound. For a while the skin has a tendency to be dry and scaly, but this can be overcome by the use of vaseline. Scarlet Red is not an antiseptic and has no place on a wound that is not clean, though some unhealthy granulating wounds appear to be benefited by the application of Scarlet Red in combination with iodoform or balsam of Peru ointment. However, one can usually secure a clean surface by the judicious use of nitrate of silver and frequent dressings. We have practiced about the following routine in using Scarlet Red :-

See that the wound is healthy. Cleanse it with boric acid solution and dry thoroughly. Apply the ointment in small quantity to the skin edges. Experience with various strengths has led us to adopt the 8% ointment. Cover the wound with sterile perforated old linen or gauze. The latter is entirely satisfactory if care is exercised in removing the dressings. This should be changed in 24 hours and sterile boric acid ointment applied. Repeat the Scarlet Red application in 24 hours. If there appears to have been any irritation of the surrounding skin, apply boric acid ointment up to about centimeter of the wound edge. We have never seen any irritation from the 8% ointment. Cleanse the wound with boric acid and salt solution at each dressing and dry. Warn the patient that the discoloration of the dressing will be due to the ointment and not to bleeding.

## Figures and Tables

**Figure f1:**
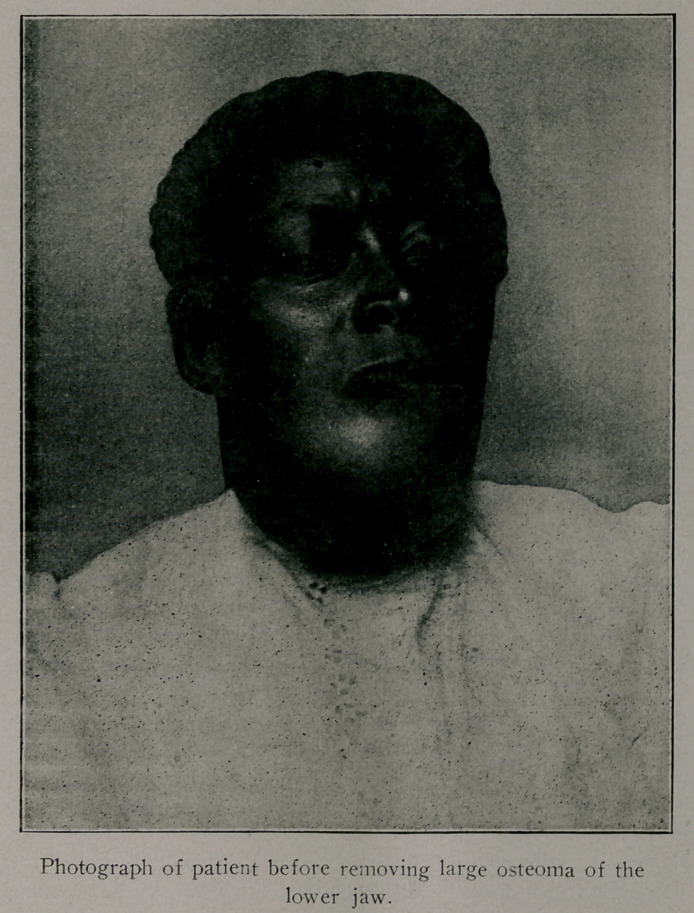


**Figure f2:**
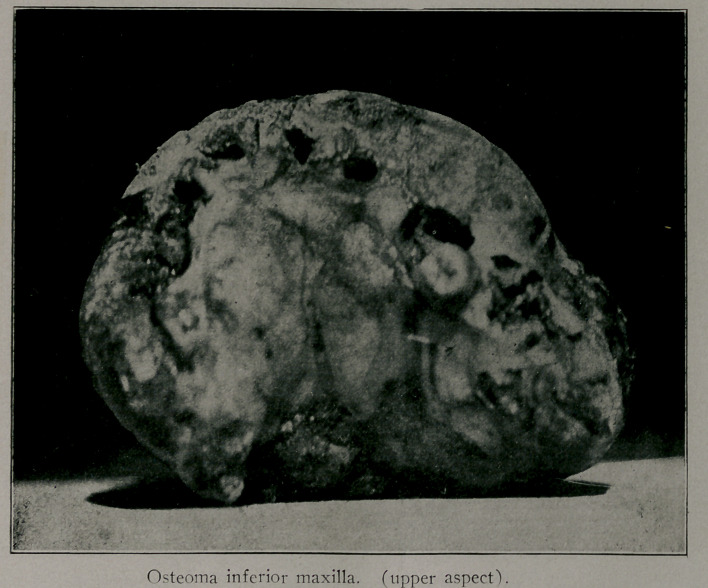


**Figure f3:**
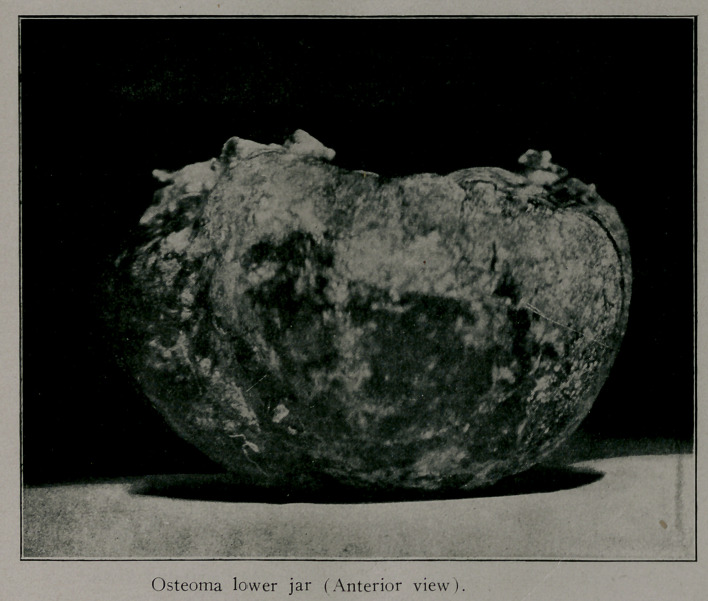


**Figure f4:**
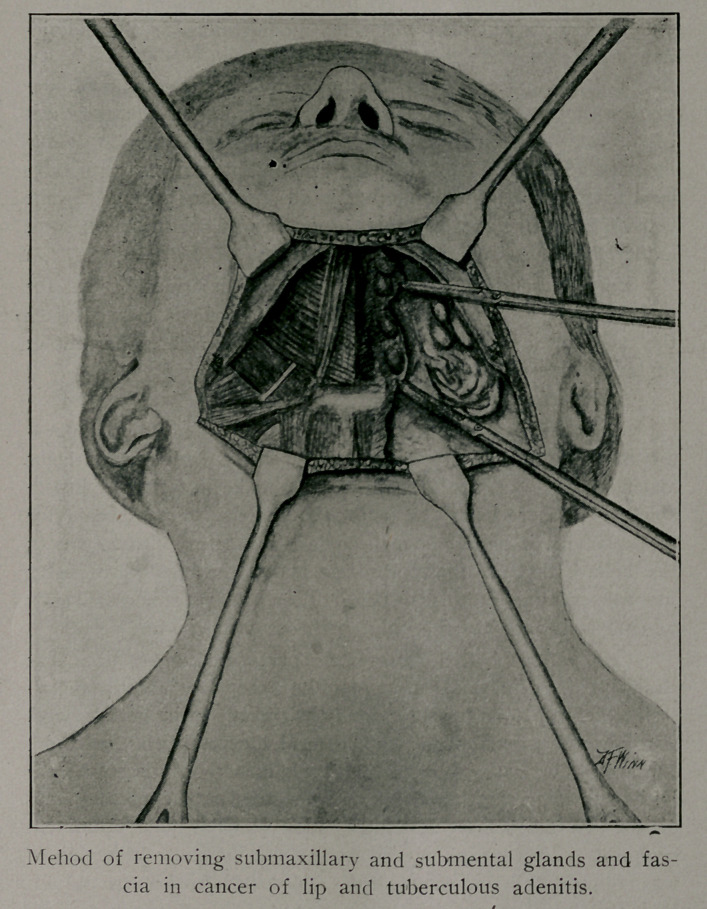


**Figure f5:**
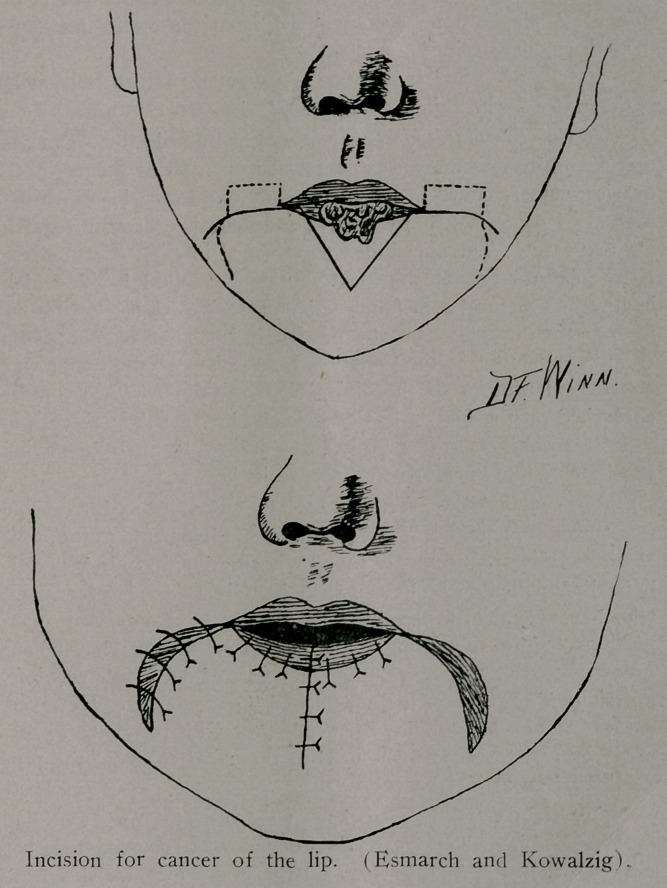


**Figure f6:**